# Serum retinol and subsequent risk of cancer.

**DOI:** 10.1038/bjc.1986.267

**Published:** 1986-12

**Authors:** N. Wald, J. Boreham, A. Bailey

## Abstract

In a prospective study of about 22,000 men attending a well person screening centre, serum samples were collected and stored. The concentration of retinol was measured in the stored serum samples from 227 men subsequently notified as having cancer and from 454 unaffected controls, matched for age, smoking history and duration of storage of the serum samples. The mean serum retinol concentration of the cancer subjects who developed cancer before the elapse of one year since the time blood was collected was significantly lower than the mean concentration of their matched controls (641 and 722 micrograms l-1 respectively, P less than 0.001). For subjects whose cancer developed one to two years after blood had been collected, the difference was less (650 and 701 micrograms l-1 respectively, P less than 0.01). For subjects whose cancer developed three or more years after blood was collected, the mean retinol level was higher than in their controls, although not statistically significantly so (694 and 663 micrograms l-1 respectively). These findings suggest that the inverse association between serum retinol and risk of cancer that was previously observed was due to low serum retinol being a metabolic consequence of cancer rather than a precursor of cancer.


*Br. J. Cancer* (1986), **54**, 957–961

### Serum retinol and subsequent risk of cancer

N. Wald[1], J. Boreham[1] & A. Bailey[2]

[1]*Department of Environmental and Preventive Medicine, St Bartholomew's Hospital Medical College, Charterhouse Square, London EC1M 6BQ, and* [2]*BUPA Medical Centre, Battle Bridge House, 300 Gray's Inn Road, London WC1X 8DU, UK.*

**Summary** In a prospective study of about 22,000 men attending a well person screening centre, serum samples were collected and stored. The concentration of retinol was measured in the stored serum samples from 227 men subsequently notified as having cancer and from 454 unaffected controls, matched for age, smoking history and duration of storage of the serum samples. The mean serum retinol concentration of the cancer subjects who developed cancer before the elapse of one year since the time blood was collected was significantly lower than the mean concentration of their matched controls (641 and 722 $\mu g l^{-1}$ respectively, $P<0.001$). For subjects whose cancer developed one to two years after blood had been collected, the difference was less (650 and 701 $\mu g l^{-1}$ respectively, $P<0.01$). For subjects whose cancer developed three or more years after blood was collected, the mean retinol level was *higher* than in their controls, although not statistically significantly so (694 and 663 $\mu g l^{-1}$ respectively). These findings suggest that the inverse association between serum retinol and risk of cancer that was previously observed was due to low serum retinol being a metabolic consequence of cancer rather than a precursor of cancer.

In an earlier paper (Wald *et al.*, 1980*a*) we reported the preliminary results from a prospective study of about 16,000 men who attended a well person screening centre. At their visit a serum sample was collected and stored. Serum retinol levels were later measured in the stored samples from the 86 men who were subsequently notified as having developed cancer and from 172 controls who were free from cancer. Low retinol levels were associated with an increased risk of cancer.

The association we observed could have arisen because low retinol levels were a metabolic consequence of the cancer or because cancer was more likely to arise in subjects who had low serum retinol levels. We concluded that the results supported the latter explanation because the mean serum retinol level in those for whom there was a suspicion of cancer when the serum was collected was not lower than in those for whom there was no evidence of cancer at the time.

Since our publication, a number of other prospective epidemiological studies have been reported on serum retinol and cancer; one yielded a similar result (Kark *et al.*, 1981), another did so only in smokers (Salonen *et al.*, 1985), but the others showed either no significant association or one of smaller magnitude (Wald *et al.*, 1984; Willett *et al.*, 1984*a,b*; Menkes & Comstock, 1984; Peleg *et al.*, 1984; Stahelin *et al.*, 1984; Nomura *et al.*, 1985; Friedman *et al.*, 1986).

To investigate the matter further we continued to make observations on the same cohort of men as well as on an additional 6,000 men and now report an examination of new data on serum retinol and cancer. We pay special attention to the results in relation to the interval between the collection of serum and the diagnosis of the cancer and compare our present results with our earlier ones.

Correspondence: N. Wald.
Received 10 May 1986.

### Methods

The design of our prospective study has been described (Wald *et al.*, 1980). About 22,000 men aged 35–64 attended the BUPA Medical Centre in London for a comprehensive medical examination between March 1975 and March 1982. Blood was collected at this examination and stored at −40°C. The National Health Service records for these men were flagged and through the assistance of the Office of Population Censuses and Surveys (OPCS) notification was received in the event of cancer or death. By April 1985, 227 men were identified as having developed cancer (subjects) excluding the 86 men who were the subjects of our preliminary study. As before two controls were selected for each of the subjects, matched on age (within 5 years), duration of storage of the serum sample (within 3 months), calendar quarter of attendance at the Medical Centre, smoking status (current smoker, ex-smoker or lifelong non-smoker) and, for current smokers, smoking habits – type of product smoked (cigarette, cigar or pipe), amount smoked (within 5 cigarettes/day, 2 cigars/day or an ounce of tobacco/week) and age started smoking (within 5 years).

© The Macmillan Press Ltd., 1986

The retinol estimations were performed in the same laboratory and by the same method as before (high pressure liquid chromatography) (Vuilleumier *et al.*, 1983). Samples were tested in three separate series with about two years between each; sera from subjects and their matched controls were always assayed in the same analytical batch. Mean retinol concentrations were standardised using the indirect method, to take account of any changes in assay performance between each series.

### Results

The mean retinol concentration for all subjects was similar to that for all controls, ignoring the interval between blood collection and diagnosis of cancer (670 and 688 $\mu g l^{-1}$ respectively). Table I shows the mean serum retinol concentration of subjects and matched controls according to the site of the cancer and the interval between blood collection and diagnosis of cancer. Particular cancer sites were analysed separately if, for each interval between blood collection and diagnosis, 5 or more men had developed cancer at that site. Other sites were grouped together. There was a statistically significant difference for those subjects whose cancer developed before the elapse of one year since the blood was collected compared with their matched controls (641 and 722 $\mu g l^{-1}$ respectively). For subjects whose cancer developed 1–2 years after blood had been collected the difference was less (650 and 701 $\mu g l^{-1}$ respectively) but still statistically significant. For subjects whose cancers developed three or more years after blood was collected, the difference was not statistically significant and the retinol level in the subjects was actually higher than that in their controls (694 and 663 $\mu g l^{-1}$ respectively). For these subjects, there was no suggestion of a difference in serum retinol between subjects and controls for cancers at any of the specified sites. Also categorisation of the data into finer time periods (between cancer developing and blood sampling) did not alter the conclusions.

Table II shows the serum retinol levels for subjects and controls from our preliminary study according to the time between collection of the blood sample and the diagnosis of cancer. (In the preliminary study, retinol levels were expressed in iu $dl^{-1}$. Here they have been converted into $\mu g l^{-1}$; 1 iu $dl^{-1}$ is equivalent to 3 $\mu g l^{-1}$). The preliminary and the second study yield consistent results (Tables II and I respectively). The inverse association between serum retinol and cancer observed in the preliminary study is evident in the second study, but only for subjects in whom the diagnosis of cancer occurred less than three years after blood was collected. A similar conclusion can be drawn from Table III which shows the number of subjects and controls according to quintile of retinol concentration in both the preliminary and the second study. There is a statistically significant inverse trend among the subjects in whom a diagnosis was made before the elapse of three years since blood was taken, but this was not the case for those diagnosed later.

### Discussion

We have confirmed the inverse association between serum retinol and cancer that we reported before (Wald *et al.*, 1980*a*) but, with the longer follow-up possible in this second study, found that the association was restricted to men who were notified as having cancer less than three years after blood was collected. This suggests that the low serum retinol levels were a metabolic consequence of the cancer rather than a precursor, even though the cancer may not have been symptomatic or clinically apparent when blood was collected.

Although in our preliminary study serum retinol levels were not materially related to the matching criteria, in this one there was a small but statistically significant decrease in serum retinol with age (736, 727, 676, 701, 678, 662 $\mu g l^{-1}$ for controls aged 35–39, 40–44, 45–49, 50–54, 55–59, 60–64 respectively). There was also a suggestion of some decrease in retinol levels with storage beyond about 5 years (688, 732, 713, 655, 658 $\mu g l^{-1}$ for samples from controls stored for 0–, 2–, 4–, 6–, 8+ years).

In the two other studies showing an inverse association between serum retinol and cancer, one (Salonen *et al.*, 1985) had a short follow-up, similar to our preliminary study and this may have explained the finding. The other study (Kark *et al.*, 1981) had a longer follow-up, but, unlike our own, the serum samples from cancer subjects had been repeatedly frozen and thawed. This was not the case with samples from the controls and the difference may have accounted for the lower retinol levels observed in subjects compared with controls.

Our results on serum retinol and cancer are similar to those of Rose and Shipley (1980), on serum cholesterol and cancer. In their study, low serum cholesterol levels were found in persons who developed cancer within two years of blood collection but not in those who developed cancer later. They also concluded that this was a metabolic consequence of the cancer. Serum cholesterol and serum retinol are known to be associated (Kark *et al.*, 1982; Marenah *et al.*, 1983) and this was evident in our study, ($r=0.12$, $P<0.01$ among the controls and $r=0.24$, $P<0.001$ among the subjects). The association suggests that they are both determined by a third factor which is itself affected by cancer, or less plausibly, that one level determines the other directly.

The results now available make it unlikely that retinol in the concentrations present in the sera of residents of developed countries had any substantial effect on the risk of developing cancer. It follows that either the lowest levels typically found in developed countries are above that which produces an observable effect or that the preventive action suggested by animal and *in vitro* experiments (Sporn *et al.*, 1984) is not apparent at concentrations naturally found in man.

We thank Dr R.M. Salkeld and Dr J.-P. Vuilleumier of Hoffmann-La Roche, Basle, Switzerland, for performing the retinol assays and for their comments, Mr P. Thompson for technical assistance, Dr J. Densem for computing assistance, Mr S. Thompson for statistical advice and Sir Richard Doll and Dr H.S. Cuckle for their comments. We also thank the Medical Research Council and the Imperial Cancer Research Fund for their financial support.

### References

FRIEDMAN, G.D., BLANER, W.S., GOODMAN, D.S., & 5 others (1986). Serum retinol and retinol binding protein levels do not predict subsequent lung cancer. *Am. J. Epidemiol.*, **123**, 781.

KARK, J.D., SMITH, A.H. & HAMES, C.G. (1982). Serum retinol and the inverse relationship between serum cholesterol and cancer. *Brit. Med. J.*, **284**, 152.

KARK, J.D., SMITH, A.H., SWITZER, B.R. & HAMES, C.G. (1981). Serum vitamin A (retinol) and cancer incidence in Evans County, Georgia. *J. Natl Cancer Inst.*, **66**, 7.

MARENAH, C.B., LEWIS, B., HASSALL, D. & 8 others. (1983). Hypocholesterolaemia and non-cardiovascular disease: Metabolic studies on subjects with low plasma cholesterol concentrations. *Brit. Med. J.*, **286**, 1603.

MENKES, M. & COMSTOCK, G. (1984). Vitamins A and E and lung cancer. *Am. J. Epidemiol.*, **120**, 491.

NOMURA, A.M.Y., STEMMERMANN, G.N., HEILBRUN, L.K., SALKELD, R.M. & VUILLEUMIER, J.P. (1985). Serum vitamin levels and the risk of cancer of specific sites in men of Japanese ancestry in Hawaii. *Cancer Res.*, **45**, 2369.

PELEG, I., HEYDEN, S., KNOWLES, M. & HAMES, C.G. (1984). Serum retinol and risk of subsequent cancer: Extension of the Evans County, Georgia, study. *J. Natl Cancer Inst.*, **73**, 1455.

ROSE, G. & SHIPLEY, M.J. (1980). Plasma lipids and mortality: A source of error. *Lancet*, **i**, 523.

SALONEN, J.T., SALONEN, R., LAPPETELAINEN, R., MAENPAA, P.H., ALFTHAN, G. & PUSKA, P. (1985). Risk of cancer in relation to serum concentrations of selenium and vitamins A and E: Matched case-control analysis of prospective data. *Brit. Med. J.*, **290**, 417.

SPORN, M.B., ROBERTS, A.B. & GOODMAN, D.S. (eds) (1984). *The Retinoids*, Vols. 1 and 2, Academic Press Inc.: Orlando.

STAHELIN, H.B., ROSEL, F., BUESS, E. & BRUBACHER, G. (1984). Cancer, vitamins, and plasma lipids: Prospective Basel study. *J. Natl Cancer Inst.*, **73**, 1463.

VUILLEUMIER, J.-P., KELLER, H.E., GYSEL, D. & HUNZIKER, F. (1983). Clinical chemical methods for the routine assessment of the vitamin status in human populations. Part I. The fat-soluble vitamins A and E, and beta-carotene. *Int. J. Vit. Nutr. Res.*, **53**, 265.

WALD, N.J., BOREHAM, J., HAYWARD, J.L. & BULBROOK, R.D. (1984). Plasma retinol, beta-carotene and vitamin E levels in relation to the future risk of breast cancer. *Br. J. Cancer*, **49**, 321.

WALD, N., IDLE, M., BOREHAM, J. & BAILEY, A. (1980*a*). Low serum-vitamin-A and subsequent risk of cancer. *Lancet*, **ii**, 813.

WALD, N.J., IDLE, M., BOREHAM, J. & BAILEY, A. (1980*b*). Inhaling habits among smokers of different types of cigarette. *Thorax*, **35**, 925.

WILLETT, W.C., POLK, B.F., UNDERWOOD, B.A. & HAMES, C.G. (1984*b*). Hypertension detection and follow-up program study of serum retinol, retinol-binding protein, total carotenoids, and cancer risk: A summary. *J. Natl Cancer Inst.*, **73**, 1459.

WILLETT, W.C., POLK, B.F., UNDERWOOD, B.A. & 6 others. (1984*a*). Relation of serum vitamins A and E and carotenoids to the risk of cancer. *N. Engl. J. Med.*, **310**, 430.


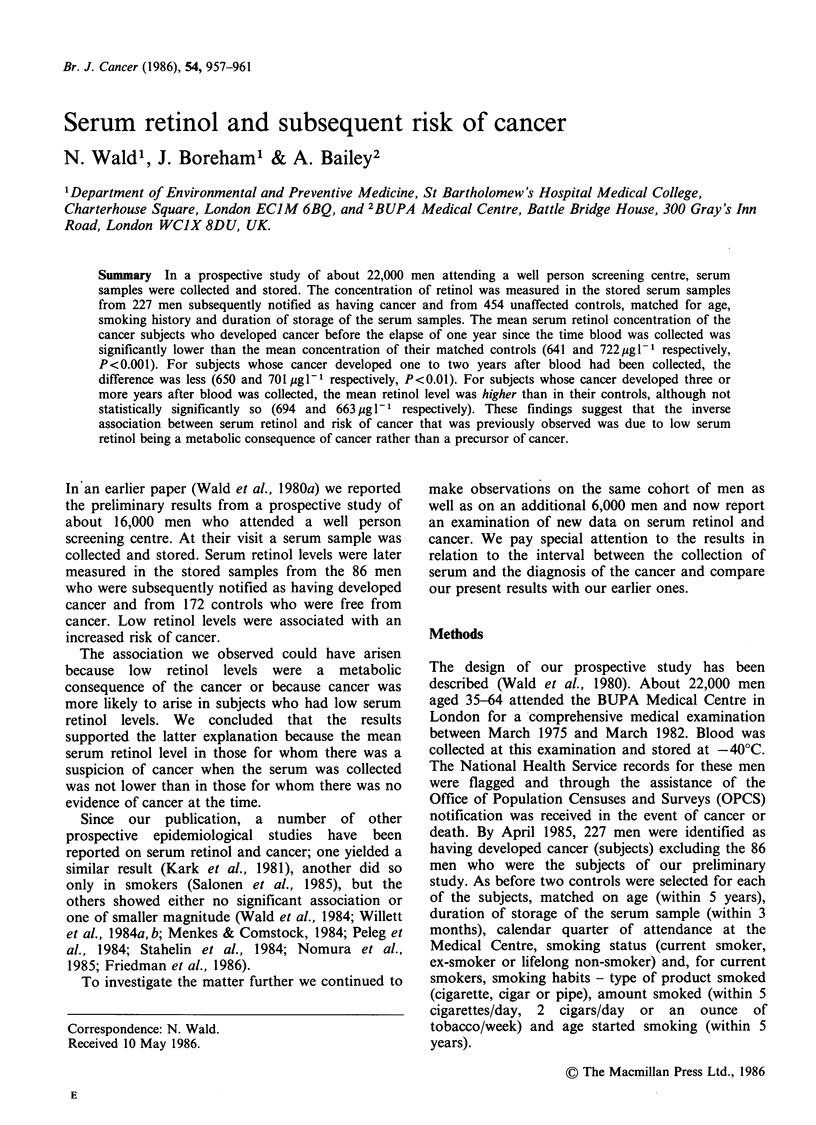

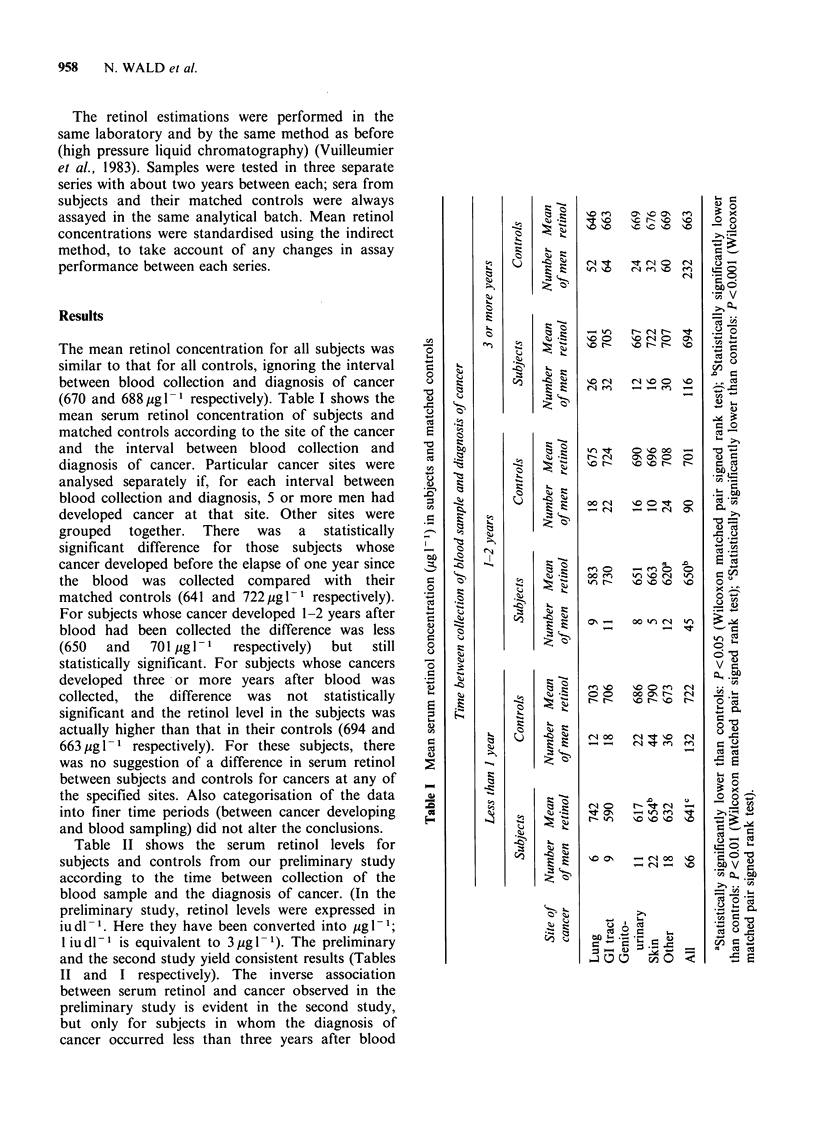

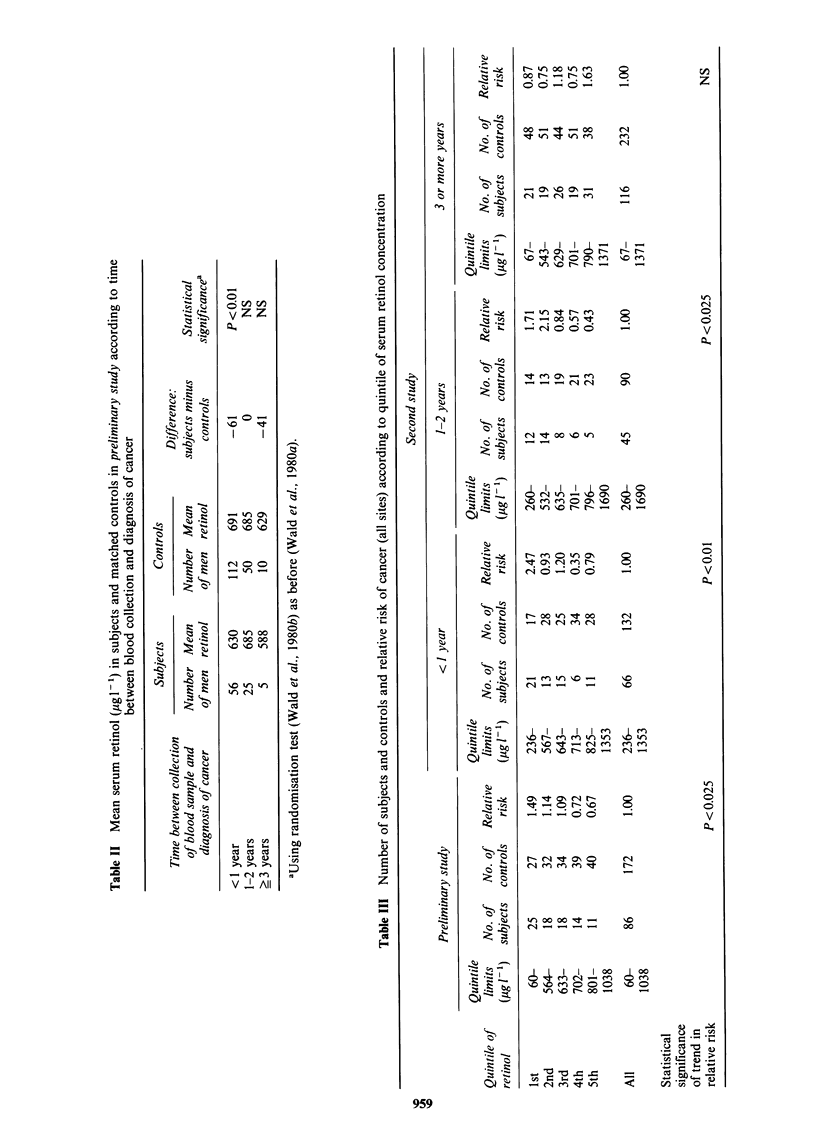

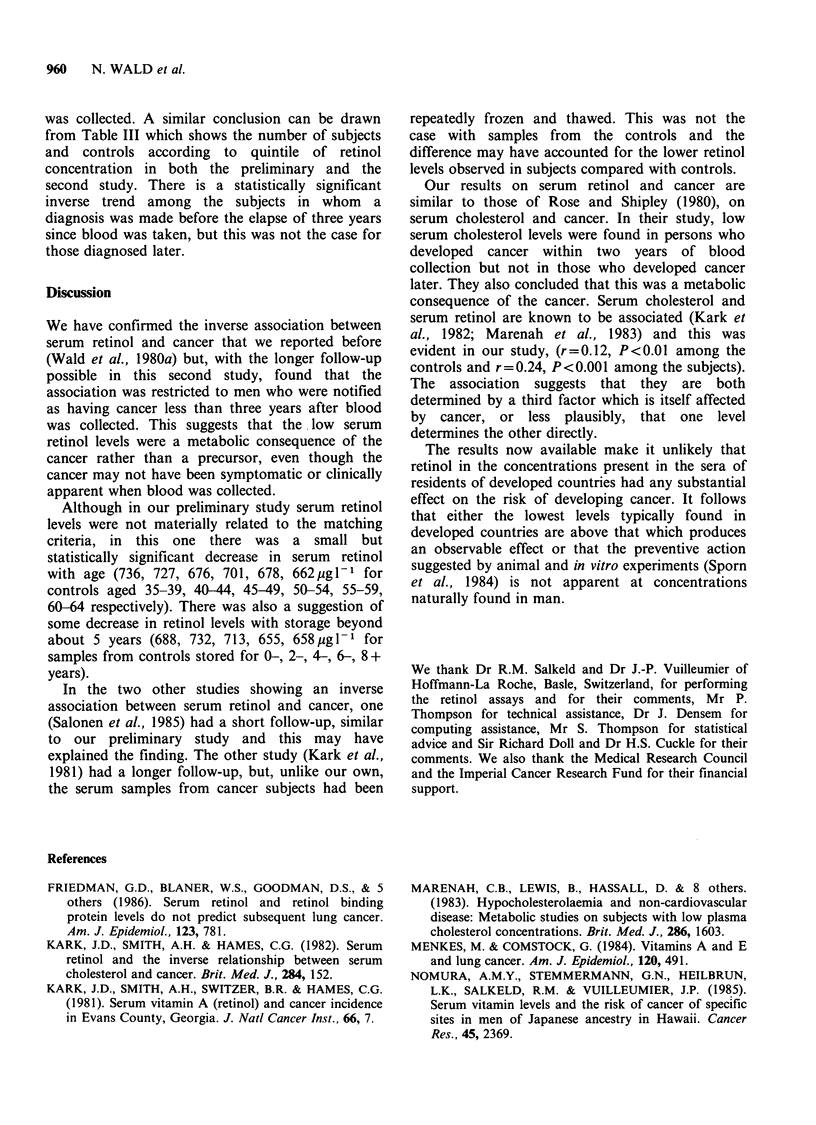

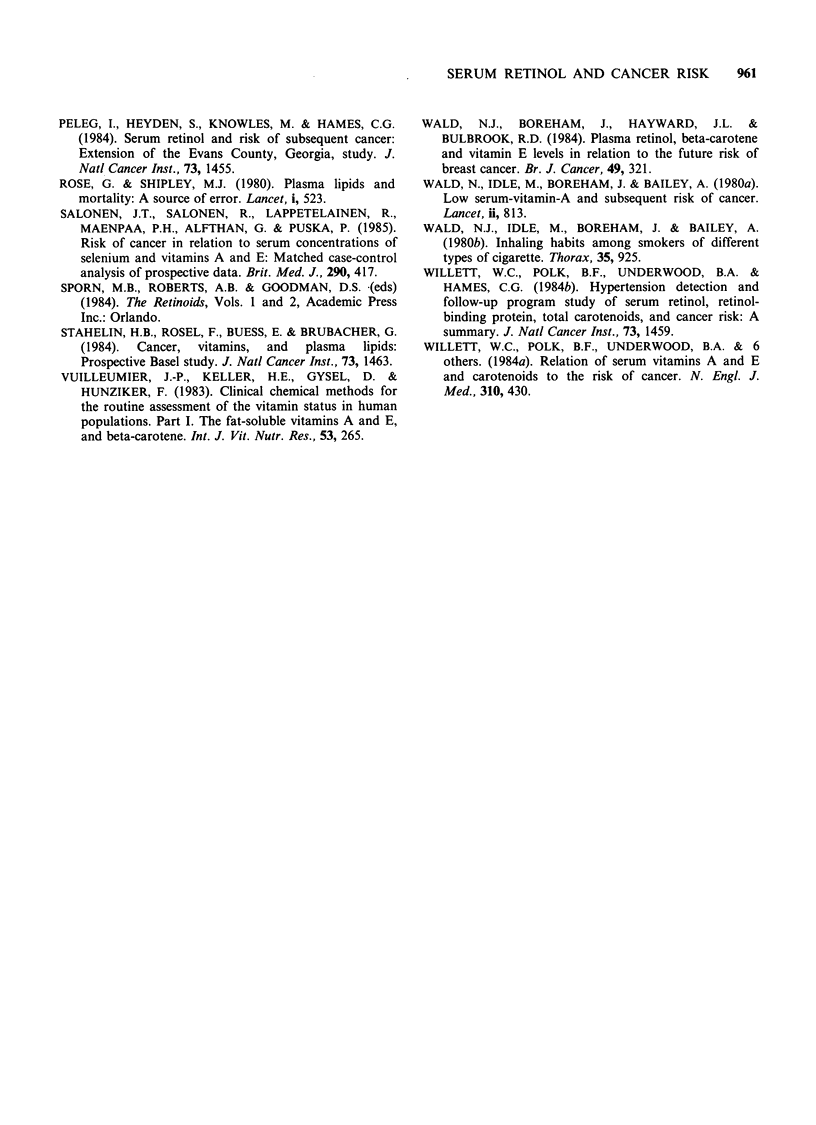


## References

[OCR_00512] Friedman G. D., Blaner W. S., Goodman D. S., Vogelman J. H., Brind J. L., Hoover R., Fireman B. H., Orentreich N. (1986). Serum retinol and retinol-binding protein levels do not predict subsequent lung cancer.. Am J Epidemiol.

[OCR_00518] Kark J. D., Smith A. H., Hames C. G. (1982). Serum retinol and the inverse relationship between serum cholesterol and cancer.. Br Med J (Clin Res Ed).

[OCR_00523] Kark J. D., Smith A. H., Switzer B. R., Hames C. G. (1981). Serum vitamin A (retinol) and cancer incidence in Evans County, Georgia.. J Natl Cancer Inst.

[OCR_00528] Marenah C. B., Lewis B., Hassall D., La Ville A., Cortese C., Mitchell W. D., Bruckdorfer K. R., Slavin B., Miller N. E., Turner P. R. (1983). Hypocholesterolaemia and non-cardiovascular disease: metabolic studies on subjects with low plasma cholesterol concentrations.. Br Med J (Clin Res Ed).

[OCR_00538] Nomura A. M., Stemmermann G. N., Heilbrun L. K., Salkeld R. M., Vuilleumier J. P. (1985). Serum vitamin levels and the risk of cancer of specific sites in men of Japanese ancestry in Hawaii.. Cancer Res.

[OCR_00547] Peleg I., Heyden S., Knowles M., Hames C. G. (1984). Serum retinol and risk of subsequent cancer: extension of the Evans County, Georgia, study.. J Natl Cancer Inst.

[OCR_00553] Rose G., Shipley M. J. (1980). Plasma lipids and mortality: a source of error.. Lancet.

[OCR_00557] Salonen J. T., Salonen R., Lappeteläinen R., Mäenpä P. H., Alfthan G., Puska P. (1985). Risk of cancer in relation to serum concentrations of selenium and vitamins A and E: matched case-control analysis of prospective data.. Br Med J (Clin Res Ed).

[OCR_00569] Stähelin H. B., Rösel F., Buess E., Brubacher G. (1984). Cancer, vitamins, and plasma lipids: prospective Basel study.. J Natl Cancer Inst.

[OCR_00574] Vuilleumier J. P., Keller H. E., Gysel D., Hunziker F. (1983). Clinical chemical methods for the routine assessment of the vitamin status in human populations. Part I: The fat-soluble vitamins A and E, and beta-carotene.. Int J Vitam Nutr Res.

[OCR_00581] Wald N. J., Boreham J., Hayward J. L., Bulbrook R. D. (1984). Plasma retinol, beta-carotene and vitamin E levels in relation to the future risk of breast cancer.. Br J Cancer.

[OCR_00592] Wald N. J., Idle M., Boreham J., Bailey A. (1980). Inhaling habits among smokers of different types of cigarette.. Thorax.

[OCR_00587] Wald N., Idle M., Boreham J., Bailey A. (1980). Low serum-vitamin-A and subsequent risk of cancer. Preliminary results of a prospective study.. Lancet.

[OCR_00597] Willett W. C., Polk B. F., Underwood B. A., Hames C. G. (1984). Hypertension detection and follow-up program study of serum retinol, retinol-binding protein, total carotenoids, and cancer risk: a summary.. J Natl Cancer Inst.

[OCR_00604] Willett W. C., Polk B. F., Underwood B. A., Stampfer M. J., Pressel S., Rosner B., Taylor J. O., Schneider K., Hames C. G. (1984). Relation of serum vitamins A and E and carotenoids to the risk of cancer.. N Engl J Med.

